# Proteolytic Processing of Nlrp1b Is Required for Inflammasome Activity

**DOI:** 10.1371/journal.ppat.1002659

**Published:** 2012-04-19

**Authors:** Bradley C. Frew, Vineet R. Joag, Jeremy Mogridge

**Affiliations:** Department of Laboratory Medicine and Pathobiology, University of Toronto. Toronto, Ontario, Canada; University of California Los Angeles, United States of America

## Abstract

Nlrp1b is a NOD-like receptor that detects the catalytic activity of anthrax lethal toxin and subsequently co-oligomerizes into a pro-caspase-1 activation platform known as an inflammasome. Nlrp1b has two domains that promote oligomerization: a NACHT domain, which is a member of the AAA+ ATPase family, and a poorly characterized Function to Find Domain (FIIND). Here we demonstrate that proteolytic processing within the FIIND generates N-terminal and C-terminal cleavage products of Nlrp1b that remain associated in both the auto-inhibited state and in the activated state after cells have been treated with lethal toxin. Functional significance of cleavage was suggested by the finding that mutations that block processing of Nlrp1b also prevent the ability of Nlrp1b to activate pro-caspase-1. By using an uncleaved mutant of Nlrp1b, we established the importance of cleavage by inserting a heterologous TEV protease site into the FIIND and demonstrating that TEV protease processed this site and induced inflammasome activity. Proteolysis of Nlrp1b was shown to be required for the assembly of a functional inflammasome: a mutation within the FIIND that abolished cleavage had no effect on self-association of a FIIND-CARD fragment, but did reduce the recruitment of pro-caspase-1. Our work indicates that a post-translational modification enables Nlrp1b to function.

## Author Summary

Inflammasomes are multi-protein complexes that respond to signals derived from microbial pathogens or damaged tissue. The function of an inflammasome is to activate pro-caspase-1, a protease that contributes to the inflammatory response by generating the cytokines IL-1β and IL-18. A common feature of inflammasomes is their ability to cluster multiple copies of pro-caspase-1 in a manner that allows inter-molecular auto-proteolysis. The Nlrp1b inflammasome assembles in response to anthrax lethal toxin by using two oligomerization regions: the NACHT domain and the FIIND-CARD region. Here, we demonstrate that the FIIND is proteolytically cleaved, but that the two fragments of Nlrp1b generated from the cleavage remain associated with one another. Cleavage within the FIIND is functionally important, however, because mutants of Nlrp1b that are not cleaved are not able to activate pro-caspase-1. Furthermore, we were able to control cleavage by inserting a heterologous protease site into Nlrp1b, which allowed us to establish that processing of Nlrp1b is required for its activity. Finally, we provide evidence that processing of Nlrp1b facilitates the recruitment of pro-caspase-1. Our work identifies a novel mechanism by which the Nlrp1b inflammasome may be regulated.

## Introduction

Inflammasomes are multi-protein complexes that facilitate the activation of pro-caspase-1 in response to pathogen associated molecular patterns (PAMPS) or endogenous danger associated molecular patterns (DAMPS). A common feature of inflammasomes is that they recruit multiple copies of pro-caspase-1, which allows auto-proteolytic processing to generate active caspase-1 that cleaves downstream targets including pro-IL-1β and pro-IL-18. There are distinct mechanisms by which inflammasomes are thought to cluster molecules of pro-caspase-1. AIM2 has a HIN domain that binds cytosolic DNA derived from viruses or intracellular bacterial pathogens; it is the binding of several molecules of AIM2 to the same fragment of cytosolic DNA that leads to the grouping of pro-caspase-1 via the ASC adaptor [1]–[3]. In contrast, NLRP3 and NLRC4 each have a NACHT domain that self-associates to assemble a pro-caspase-1 activation platform after their respective triggers release auto-inhibitory intra-molecular interactions. Formation of the NLRP3 inflammasome has been proposed to occur in response to lysosomal permeabilization or reactive oxygen species generation [4]–[6], although recent evidence suggests that reactive oxygen species may serve only to induce the expression of NLRP3 [7]. The NLRC4 inflammasome detects bacterial flagellin and secretion system components [8], [9]. Human NLRP1 and the murine homolog, Nlrp1b, have NACHT domains as well as a Function to Find Domain (FIIND) that facilitate self-association [10], [11]. Nlrp1b indirectly senses the proteolytic activity of anthrax lethal toxin (LeTx) and there is some evidence that NLRP1 may detect peptidoglycan [12]–[16].

LeTx is comprised of a protease, lethal factor (LF), and a second component, protective antigen (PA), which binds mammalian cells and translocates LF to the cytosol [17], [18]. The proteolysis of multiple members of the MAPKK family by LF results in downregulation of the ERK, p38 and JNK signaling pathways. Interference of these pathways explains many of the pathogenic effects of LeTx, such as the inhibition of cytokine expression and chemotaxis, but there is no clear link between inactivation of these targets and activation of the Nlrp1b inflammasome [19]. Activation of Nlrp1b by LeTx causes macrophages to undergo a form of caspase-1 dependent cell death known as pyroptosis [20]. Interestingly, induction of pyroptosis in macrophages promotes the survival of mice infected with *Bacillus anthracis* by initiating IL-1β signaling and neutrophil recruitment [21], [22].

**Figure 1:**
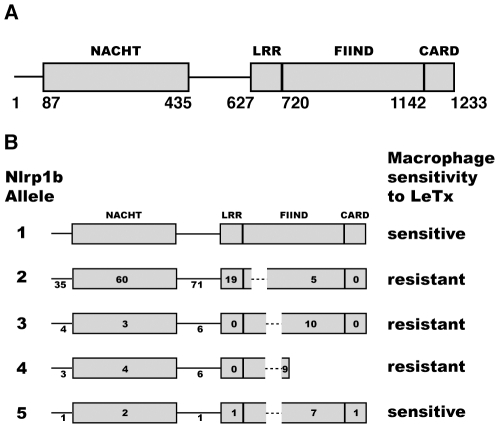
Schematic domain structure of Nlrp1b. (A) Nlrp1b1 NACHT domain (residues 87–435), LRR domain (residues 627–719), FIIND (residues 720–1141) and CARD domain (residues 1142–1233) are shown. (B) Comparison of Nlrp1b alleles. Numbers refer to the amino acid differences compared to allele 1 in each region of the protein. Dashed lines indicate deleted sequences.

There are five alleles of Nlrp1b. Murine macrophages that express either allele 1 or 5 are susceptible to LeTx-mediated pyroptosis, but those that express allele 2, 3, or 4 are resistant [13]. The alleles are highly polymorphic: amino acid differences are found within the NACHT domain, the leucine rich repeat (LRR) domain, the FIIND, the caspase recruitment domain (CARD), as well as inter-domain regions (Figure 1). Although there has been little work done to examine the differences between the alleles, we previously developed a reconstituted cell-based system and confirmed that LeTx activated Nlrp1b allele 1 (Nlrp1b1, AAZ40509.1), but not Nlrp1b allele 3 (Nlrp1b3, AAZ40520.1) – there is a functional difference(s) between these proteins because they were expressed at similar levels [11]. We also noted in immunoblots that expression of Nlrp1b1 yielded two products, which could be interpreted as a cleavage event occurring within the protein, whereas Nlrp1b3 yielded only one product [11]. Recently, the human FIIND has been demonstrated to undergo autoproteolysis [23]. D'Osualdo and colleagues detected sequence similarity between the FIIND and the ZU5-UPA domain found in the autoproteolytic protein PIDD, which is activated by DNA damage. In both NLRP1 and PIDD, it is thought that a catalytic serine attacks a strained segment of the polypeptide backbone to generate two protein fragments that remain associated. Autoproteolysis of PIDD affects downstream signaling [24], but the consequence of NLRP1 autoproteolysis is not known.

Here, we have studied the FIIND of Nlrp1b. We made a series of truncation mutants to identify the minimal region of the FIIND that can drive oligomerization of the CARD domain and activation of pro-caspase-1. A multiple alanine substitution mutant of this Nlrp1b1 fragment impaired self-association and pro-caspase-1 activation. The same mutation abolished function of full-length Nlrp1b1 and prevented cleavage within the FIIND, so we hypothesized that cleavage of Nlrp1b is required for its function. Comparison of the Nrlp1b1 and Nlrp1b3 sequences allowed us to identify a single amino acid that determines the difference in their susceptibilities to cleavage. We demonstrated that cleavage is required for activity by introducing a heterologous TEV protease site into a cleavage-deficient mutant of Nlrp1b1 and showing that activation required co-expression of TEV protease. Finally, we provide evidence that cleavage of Nlrp1b1 does not facilitate its self-association, but does enhance the recruitment of pro-caspase-1.

## Results

### Nlrp1b1_1100–1233_ self-associates and activates pro-caspase-1

**Figure 2:**
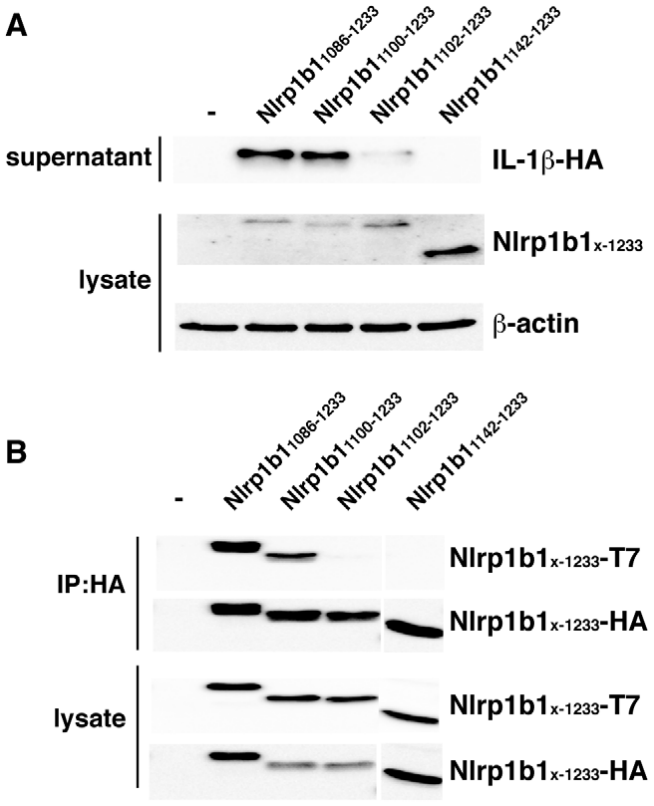
Nlrp1b1_1100–1233_ self-associates and actives pro-caspase-1. (A) HT1080 cells were co-transfected with Nlrp1b1 truncation constructs, pcDNA3-pro-caspase-1-FLAG and pcDNA3-pro-IL-1β-HA as indicated. After 24 h, cells were lysed and lysates were probed for T7-tagged Nlrp1b truncation mutants and β-actin by immunoblotting; supernatants were immunoprecipitated with anti-HA antibodies and then probed for HA-tagged IL-1β. (B) HT1080 cells were transfected with pcDNA3-His_6_-Nlrp1b1-HA and pcDNA3-His_6_-Nlrp1b1-T7 tagged Nlrp1b1 truncation constructs as indicated. Cells were lysed 24 h after transfection, and proteins were immunoprecipitated using anti-HA antibody, followed by immunoblotting with anti-T7 antibody and anti-HA antibody. Blots are representative of three independent experiments.

Previous work demonstrated that Nlrp1b1_1086–1233_ activates pro-caspase-1 in the absence of LeTx [11]. To identify the smallest constitutively active fragment of Nlrp1b1, we tested various Nlrp1b1 truncation mutants for their potential to activate pro-caspase-1 by reconstituting the Nlrp1b1 inflammasome in HT1080 fibroblasts. Various Nlrp1b1 truncation mutants were transfected into HT1080 cells along with pro-caspase-1 and pro-IL-1β and then the supernatants were probed for HA-tagged IL-1β by immunoblotting. In agreement with previous findings, IL-1β was detected in supernatants of cells expressing Nlrp1b1_1086–1233_, which contains part of the FIIND plus the CARD domain, but not the CARD domain alone, Nlrp1b1_1142–1233_ (Figure 2A). Nlrp1b1_1100–1233_ induced IL-1β secretion, whereas Nlrp1b1_1102–1233_ exhibited little activity. These results indicate, therefore, that Nlrp1b1_1100–1233_ is the smallest fragment of Nlrp1b1 that can optimally activate pro-caspase-1.

We next performed co-immunoprecipitation experiments to test self-association of the Nlrp1b1 truncation mutants. HT1080 cells were transfected with His_6_-Nlrp1b1-HA and His_6_-Nlrp1b1-T7 vectors containing the truncation mutants Nlrp1b1_1086–1233_, Nlrp1b1_1100–1233_, Nlrp1b1_1102–1233_ and Nlrp1b1_1142–1233_. HA-tagged proteins were immunoprecipitated and associated T7-tagged proteins were detected by immunoblotting (Figure 2B). Nlrp1b1_1086–1233_ and Nlrp1b1_1100–1233_ self-associated, but Nlrp1b1_1102–1233_ and Nlrp1b1_1142–1233_ did not (Figure 2B). These data support an induced-proximity model in which the self-association of Nlrp1b1 is required to activate pro-caspase-1.

### A multiple substitution mutation impairs Nlrp1b1 cleavage and activity

**Figure 3:**
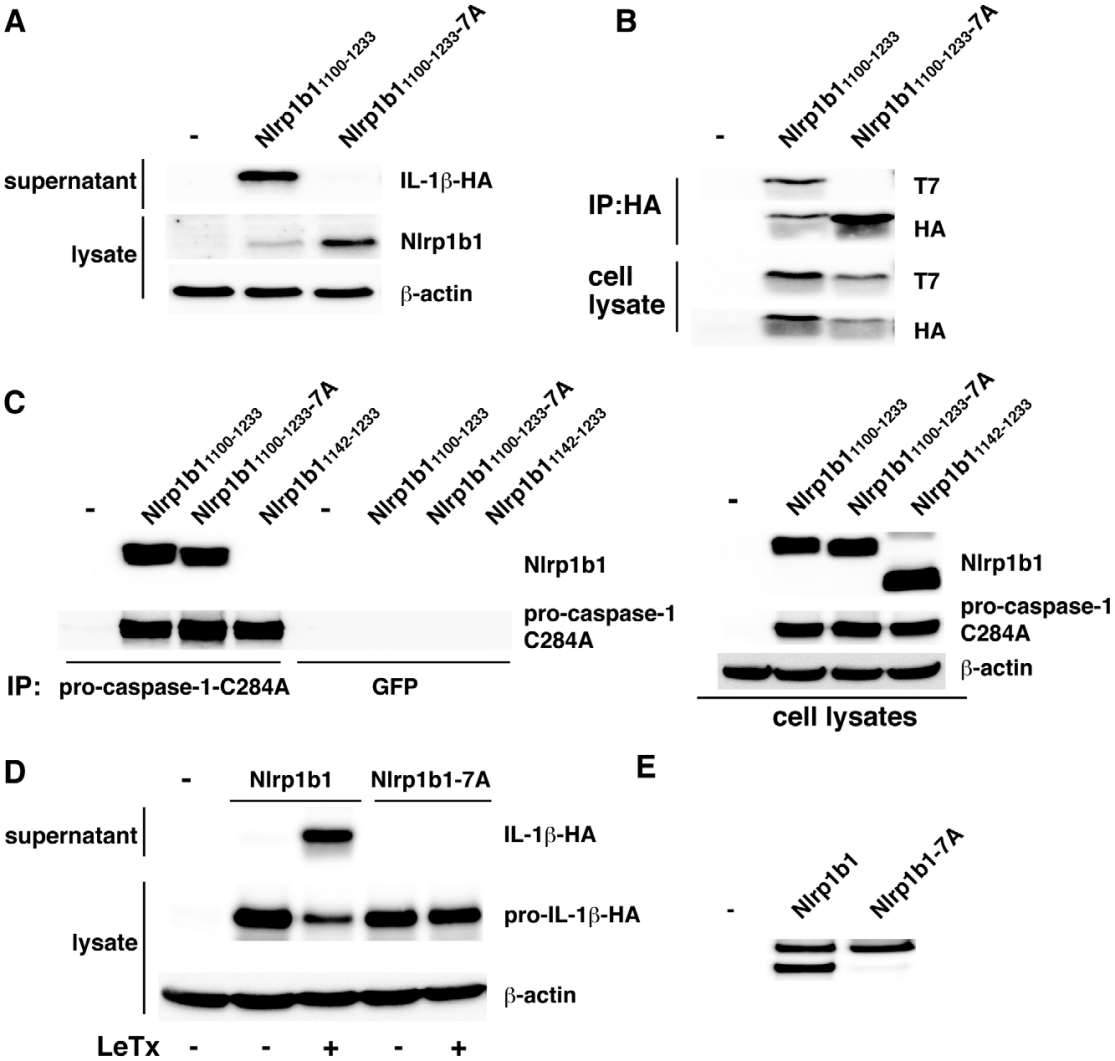
Mutation of amino acids 1100–1106 impairs FIIND self-association, FIIND cleavage, and Nlrp1b1 activity. (A) Nlrp1b1_1100–1233_ or Nlrp1b1_1100–1233_-7A (containing alanine substitution mutations of amino acids 1100–1106) was expressed with pro-caspase-1 and pro-IL-1β-HA in HT1080 cells. Cells were lysed 24 h after transfection. Cell lysates were probed for T7-tagged Nlrp1b1 constructs and for β-actin by immunoblotting; supernatants were immunoprecipitated with anti-HA antibodies and then probed for HA-tagged IL-1β. (B) T7-tagged and HA-tagged Nlrp1b1_1100–1233_ or Nlrp1b1_1100–1233_-7A were expressed in HT1080 cells. HA-tagged proteins were immunoprecipitated from cell lysates and the immunprecipitates were probed for T7- and HA-tagged proteins. (C) HA-tagged Nlrp1b1_1100–1233_, Nlrp1b1_1100–1233_-7A and Nlrp1b1_1142–1233_ were co-expressed with pro-caspase-1-C284A-T7. Immunoprecipitations were performed with anti-T7 or control anti-GFP antibodies and immunoblotted for HA and T7 epitopes. Cell lysates (right panel) were immunoblotted as indicated. (D) Nrlp1b1 and Nrlp1b1-7A were expressed with pro-caspase-1 and pro-IL-1β-HA in HT1080 cells. Cells were treated with LeTx for 3 h and supernatants were probed for IL-1β-HA as above. (E) Cell lysates from (D) were probed for Nlrp1b1. Blots are representative of three independent experiments.

We next substituted residues _1100_EIKLQIK_1106_ to alanine in Nlrp1b1_1100–1233_ and found that this mutant, Nlrp1b1_1100–1233_-7A, was impaired in its ability to self-associate and activate pro-caspase-1 (Figure 3A, B). The mutation did not, however, affect binding of catalytically inactive pro-caspase-1-C284A, which demonstrates that Nlrp1b1_1100–1233_-7A was not grossly misfolded (Figure 3C). Notably, Nlrp1b1_1100–1233_ and Nlrp1b1_1100–1233_-7A bound pro-caspase-1-C284A, whereas the CARD domain alone (Nlrp1b1_1142-123_) did not, indicating that a region N-terminal to the CARD domain facilitates the recruitment of pro-caspase-1. The multiple alanine substitution mutation was also introduced into full-length Nlrp1b1; Nlrp1b1-7A was not able to active pro-caspase-1 in response to LeTx (Figure 3D). Interestingly, Nlrp1b1-7A appeared as a single band in an immunoblot in contrast to the two bands that are observed upon expression of wild-type Nlrp1b1 (Figure 3E). These results suggest a relationship between self-association of the FIIND, cleavage of the FIIND, and activation of pro-caspase-1.

### Endogenous Nlrp1b1 is cleaved in murine macrophages

**Figure 4:**
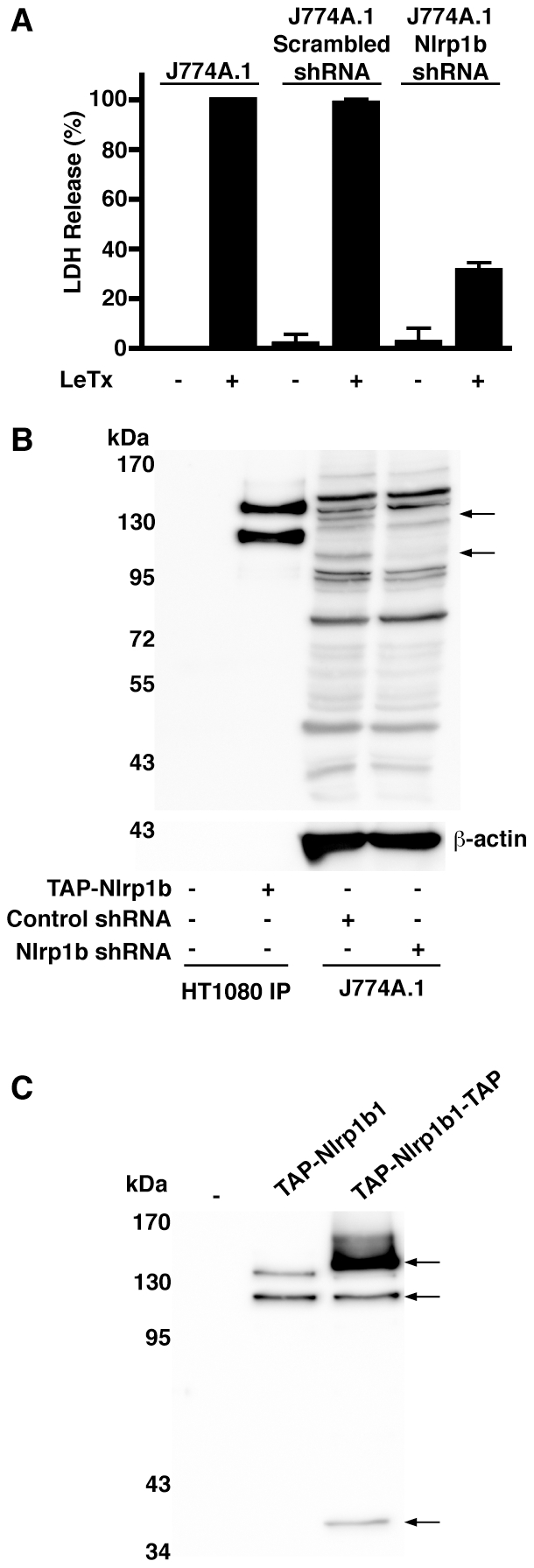
Nlrp1b1 is cleaved within the FIIND. (A) Untransduced J774A.1 cells, or J774A.1 cells stably expressing scrambled shRNA or Nlrp1b shRNA were treated with LeTx (10^−9^ M LF and 10^−8^ M PA) for 4 h and cell supernatants were assayed for LDH activity as an indication of cell death. Error bars indicate SEM of three independent experiments. (B) HT1080 cells were either transfected with pNTAP empty vector or pNTAP-Nlrp1b1. Approximately 24 h after transfection, cells were lysed and TAP-tagged proteins were precipitated with streptavidin resin. Precipitated TAP-tagged proteins and lysates from J774A.1 cells stably expressing either scrambled shRNA or Nlrp1b shRNA were subjected to immunoblotting using an anti-Nlrp1b1 antibody. Arrows indicate two bands present in control, but not in Nlrp1b1 knockdown lysates. (C) HT1080 cells were transfected with either pNTAP empty vector, pNTAP-Nlrp1b1, or pNTAP-Nlrp1b1-TAP. Approximately 24 h following transfection, cells were lysed and TAP-tagged proteins were precipitated with streptavidin resin and immunoblotted using an antibody directed against the TAP tag. Arrows indicate full-length Nlrp1b1 and two cleaved fragments of Nlrp1b1.

We sought to determine if endogenous Nlrp1b1 is proteolytically processed. First, we knocked down Nlrp1b1 in the murine macrophage cell line J774A.1 and assayed for cell death in response to LeTx. J774A.1 cells that were stably expressing Nlrp1b1 shRNA showed considerable protection from LeTx-induced cell death compared to cells expressing control shRNA, indicating that the knockdown was functionally effective (Figure 4A). We next probed these lysates with an antibody raised against the N-terminus of Nlrp1b1. Although the Nlrp1b1 antibody detects several proteins non-specifically, comparing lysates made from J774A.1 cells stably expressing either control shRNA or Nlrp1b shRNA demonstrated that the knockdown of Nlrp1b1 led to the disappearance of two bands (Figure 4B). These bands had slightly lower molecular weights than the bands corresponding to transfected TAP-tagged Nlrp1b1, which is predicted to be 8 kDa larger than endogenous Nlrp1b1. That the difference in molecular weight is similar between the two endogenous bands and the two TAP-tagged bands suggests that endogenous Nlrp1b1 is cleaved at the same location as transfected Nlrp1b1.

### Cleavage of Nlrp1b1 occurs in the FIIND

Because the TAP tag antibody and the Nlrp1b antibody both detect epitopes at the N-terminus of Nlrp1b, the two bands observed on the immunoblots likely correspond to full-length Nlrp1b1 and the N-terminal fragment of cleaved Nlrp1b1. To detect the C-terminal fragment, we generated an Nlrp1b1 construct with an additional TAP tag at the C-terminus. As predicted, expression of this doubly tagged protein yielded three bands: full-length Nlrp1b1, the N-terminal fragment, and the C-terminal fragment (Figure 4C). Judging by the molecular weight of the C-terminal fragment (estimated to be ∼30 kDa without the TAP tag), cleavage of Nlrp1b1 occurs in the FIIND.

### Nlrp1b1-V988D is not cleaved and is not responsive to LeTx

**Figure 5:**
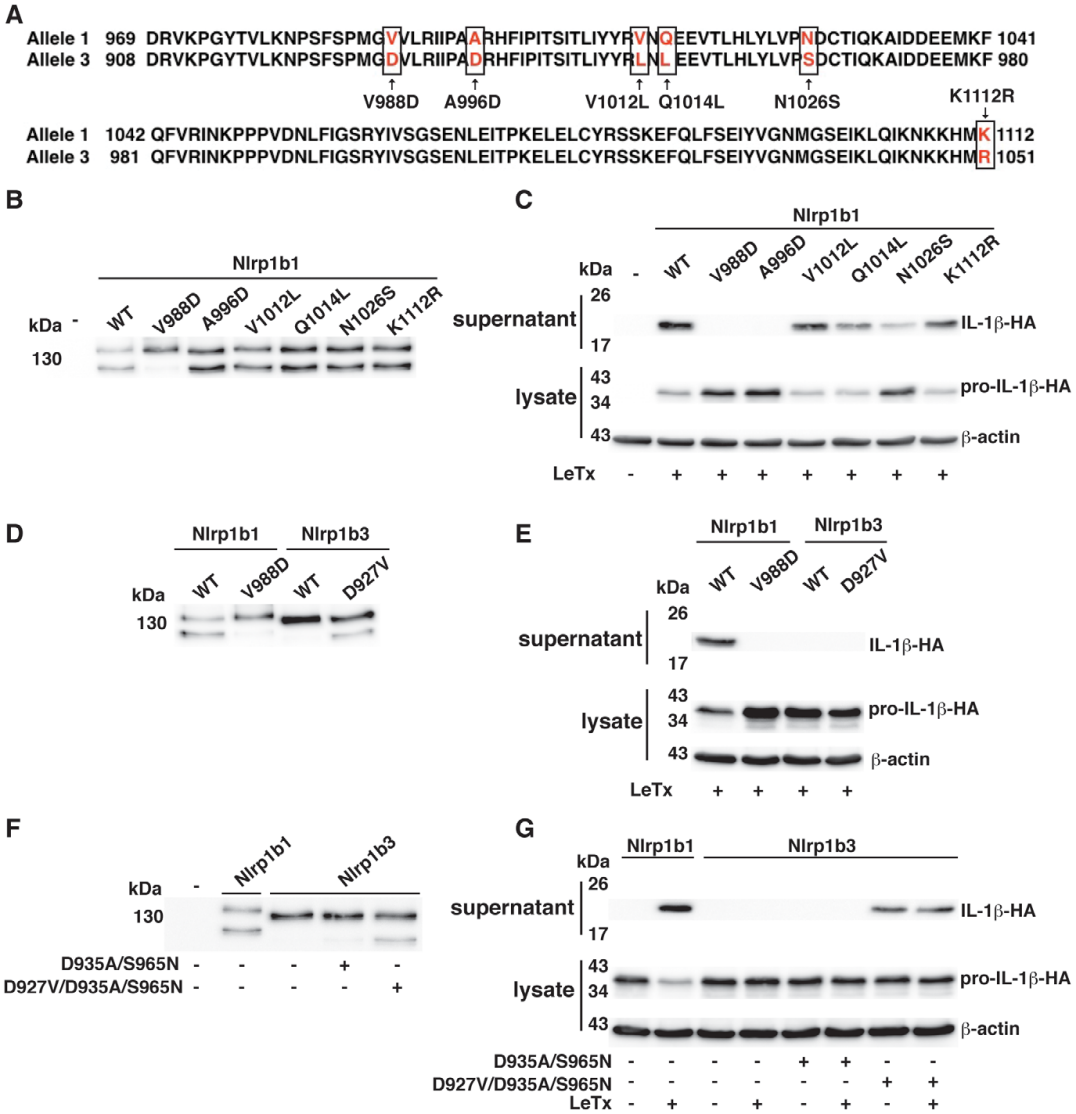
The V988D mutation in Nlrp1b1 eliminates both cleavage and inflammasome activity. (A) Alignment of allele 1 with allele 3 from amino acid 969 to 1112 of Nlrp1b1. Highlighted amino acid differences use numbering from allele 1. (B, D, and F) HT1080 cells were transfected with pNTAP plasmids encoding the indicated Nlrp1b proteins. Approximately 24 h following transfection, cells were lysed and TAP-tagged proteins were precipitated with streptavidin resin and immunoblotted using an Nlrp1b antibody. (C, E, and G) Cells were transfected with pNTAP plasmids encoding the indicated Nlrp1b constructs, as well as with pcDNA3-pro-caspase-1-T7 and pcDNA3-pro-IL-1β-HA. Approximately 24 h after transfection, cells were treated with LeTx (10^−8^ M LF and 10^−8^ M PA) for 3 h. Cell lysates were collected and probed for HA-tagged pro-IL-1β and β-actin; cell supernatants were collected and immunoprecipitated with anti-HA antibodies and probed for HA-tagged IL-1β by immunoblotting. Blots are representative of three independent experiments.

Based on the size of the C-terminal fragment and on the observation that Nlrp1b1 is cleaved whereas Nlrp1b3 is not cleaved, we speculated that an amino acid difference(s) between Nlrp1b1 and Nlrp1b3 in the region near amino acids 970–1120 accounts for the lack of cleavage of Nlrp1b3. Six amino acid differences were found near this region (Figure 5A), so we generated 6 single swap mutants of Nlrp1b1. Of the 6 mutations, only the V988D mutation resulted in a loss of cleavage of Nlrp1b1 (Figure 5B), suggesting that this difference between the two proteins is responsible for the lack of cleavage of Nlrp1b3. Notably, this mutation also resulted in a complete loss of inflammasome activity in response to LeTx, as assessed by the loss of mature IL-1β in the cell supernatant (Figure 5C). Several other mutants (most markedly A996D and N1026S) resulted in decreased inflammasome activity without affecting cleavage of Nlrp1b1. These data suggest that the non-responsiveness of Nlrp1b3 to LeTx is not only a consequence of it not being cleaved.

To determine if a single mutation could lead to the cleavage of Nlrp1b3, a mutant containing the reverse of the V988D mutation, Nlrp1b3-D927V, was made (the difference is numbering is due to an insertion in Nrlp1b1). Nlrp1b3-D927V was cleaved, but it was not able to activate pro-caspase-1 in response to LeTx (Figure 5D, E). In an attempt to generate a functional version of Nlrp1b3, we introduced swap mutations based on the mutations that impaired function of Nrlp1b1 (A996D and N1026S). Nlrpb3-D935A/S965N did not exhibit any activity, but Nlrp1b3-D927V/D935A/S965N was cleaved and displayed activity (Figure 5F, G). This triple mutant was constitutively active, presumably because the mutations also interfere with auto-inhibition of the protein.

### Cleavage of Nlrp1b1 affects its activity

**Figure 6:**
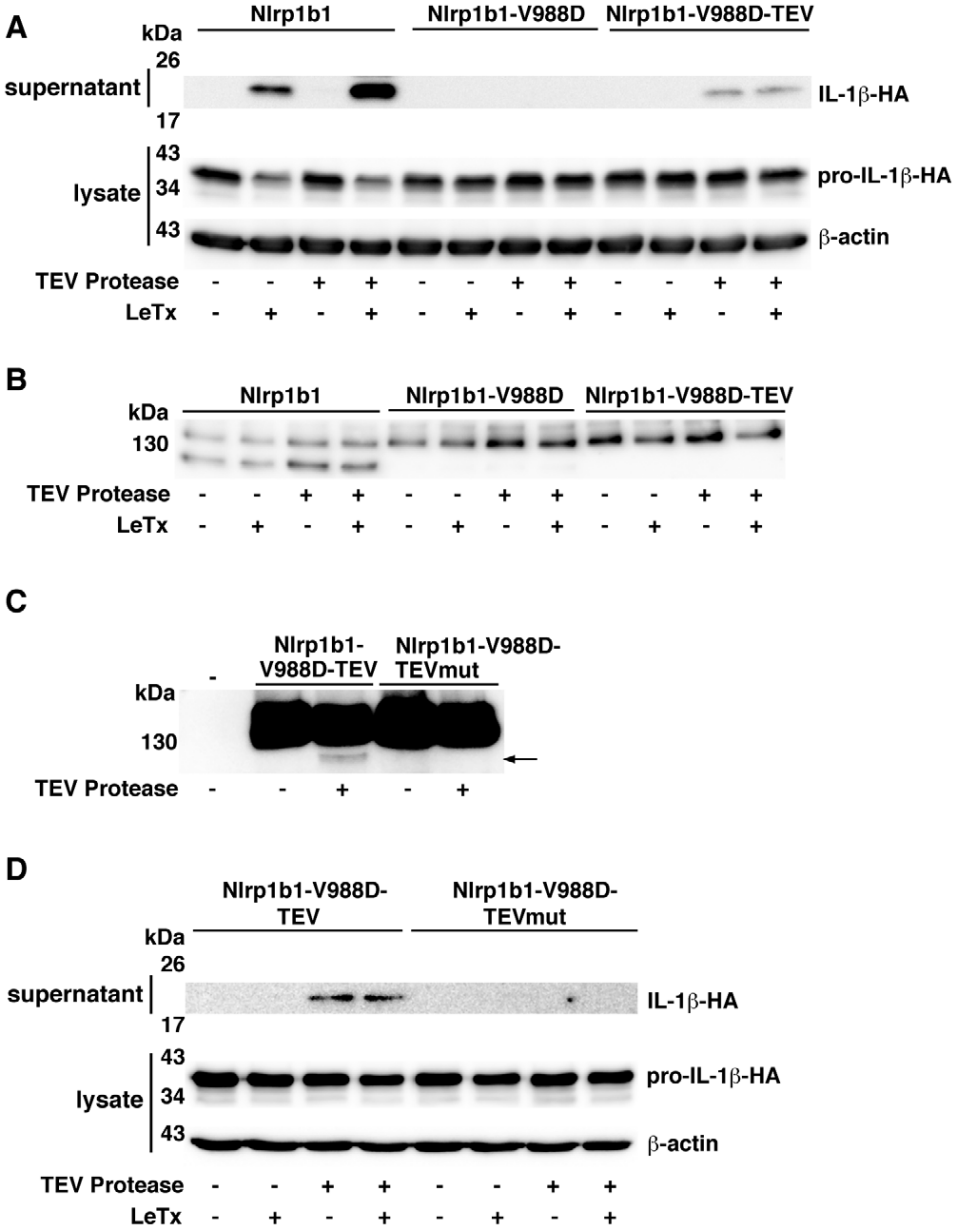
Restoration of cleavage in the Nlrp1b1 V988D mutant rescues inflammasome activity. (A and D) Cells were transfected with pcDNA3-pro-IL-1β-HA and pcDNA3-pro-caspase-1-T7 in addition to the indicated combinations of pNTAP plasmids encoding Nlrp1b proteins and pcDNA3-TEV-protease-T7. Approximately 24 h after transfection, cells were treated with LeTx for 3 h. Cell lysates were collected and probed for HA-tagged pro-IL-1β and β-actin; cell medium was collected and immunoprecipitated with anti-HA antibodies and probed for HA-tagged IL-1β by immunoblotting. (B) HT1080 cells were transfected with pNTAP plasmids encoding Nlrp1b proteins, pcDNA3-pro-IL-1β-HA, and pcDNA3-TEV-protease-T7 as indicated. Approximately 24 h after transfection, cells were treated with LeTx (10^−8^ M LF and 10^−8^ M PA) for 3 h. Cells were lysed and TAP-tagged proteins were precipitated with streptavidin resin and immunoblotted using an Nlrp1b antibody. (C) Cells were transfected with pNTAP plasmids encoding the indicated Nlrp1b proteins and pcDNA3-TEV-protease-T7 as indicated. Approximately 24 h following transfection, cells were lysed and TAP-tagged proteins were precipitated with streptavidin resin and immunoblotted using an Nlrp1b antibody. Arrow indicates cleavage product. Blots are representative of three independent experiments.

We next sought to determine whether proteolytic processing of Nlrp1b1 is important for its activity by introducing a heterologous protease site whose cleavage could be controlled. A cleavage site for the tobacco etch virus (TEV) NIa protease was inserted between amino acids 981 and 982 of Nlrp1b1-V988D. Importantly, TEV protease has high sequence specificity and is well tolerated by mammalian cells [25]. Nlrp1b1, Nlrp1b1-V988D, and Nlrp1b1-V988D-TEV were each co-expressed with pro-caspase-1 and pro-IL-1β. As expected, LeTx caused the release of IL-1β by cells that expressed Nlrp1b1 regardless of whether TEV protease was expressed (Figure 6A). TEV protease did not cause the release of IL-1β in the absence of LeTx. Nlrp1b1-V988D was not functional in any of the conditions tested, whereas Nlrp1b1-V988D-TEV activated pro-caspase-1, but only in the presence of TEV protease. That TEV protease was able to promote pro-caspase-1 activation by Nlrp1b1-V988D-TEV in the absence of LeTx indicated that TEV cleavage overrides auto-inhibition of Nlrp1b1.

Cleavage of Nlrp1b1-V988D-TEV was not observed under conditions similar to those used to test activity (with the exception that the catalytically inactive mutant pro-caspase-1-C284A was used in order to prevent release of inflammasome components from the cell), indicating that only a small fraction of Nlrp1b1-V988D-TEV may have been cleaved (Figure 6B). To confirm that direct cleavage of Nlrp1b-V988D-TEV by TEV protease was responsible for the observed activity, rather than an indirect effect of the protease, a TEV-site mutant was generated. Nlrp1b1-V988D-TEVmut has a Q to H mutation in the consensus sequence ENLYFQS, which has been shown to prevent cleavage by TEV protease [26]. Because cleavage by TEV protease could not be visualized under the conditions used to test inflammasome activation (Figure 6B), 5 times as much plasmid encoding TEV protease was transfected with the Nlrp1b1 constructs. Under these conditions, a small amount of cleaved product of Nlrp1b1-V988D-TEV was observed, whereas no cleavage of Nlrp1b1-V988D-TEVmut was detected (Figure 6C). Nlrp1b1-V988D-TEV exhibited activity when co-expressed with TEV protease, but Nlrp1b1-V988D-TEVmut did not (Figure 6D). Cumulatively, these results provide strong evidence that cleavage of Nlrp1b1 is important for its function.

### Cleaved fragments of Nlrp1b1 remain associated

**Figure 7:**
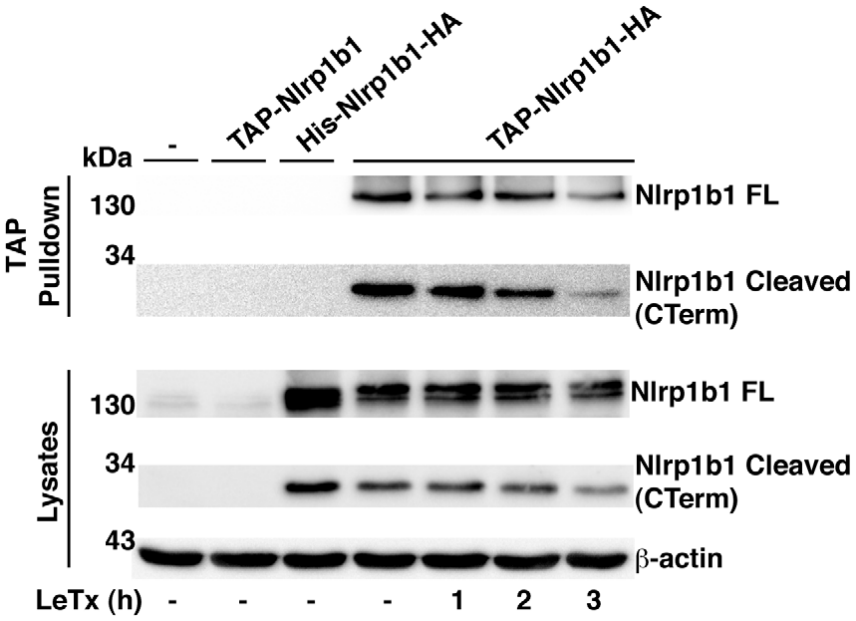
The cleaved products of Nlrp1b1 remain associated. (A) HT1080 cells were transfected with the indicated Nlrp1b1 plasmids. Approximately 24 h following transfection, cells were treated with LeTx (10^−8^ M LF and 10^−8^ M PA) for the indicated times. Cells were lysed and TAP-tagged proteins were precipitated with streptavidin resin. Proteins from the precipitates and the lysates were immunoblotted with antibodies against HA and β-actin. Blots represent three independent experiments.

We next wanted to determine whether the N-terminal and C-terminal fragments of Nlrp1b1 are associated in the auto-inhibited state and if activation of Nlrp1b1 by LeTx causes the C-terminal fragment to be released as an activated moiety. Nlrp1b1 was expressed with an N-terminal TAP tag and a C-terminal HA tag. When Nlrp1b1 was precipitated using the TAP tag, the C-terminal fragment co-precipitated (Figure 7). Treatment of cells with LeTx for 1–2 h did not reduce the amount of C-terminal fragment precipitated. Treatment of cells with LeTx for 3 h slightly reduced the amount of the C-terminal fragment precipitated, although this was because less was present in the lysate possibly as a result of inflammasome secretion. Thus, LeTx does not cause cleaved Nlrp1b1 to dissociate.

### The V988D mutation reduces the ability of Nlrp1b1_720–1233_ to associate with pro-caspase-1

**Figure 8:**
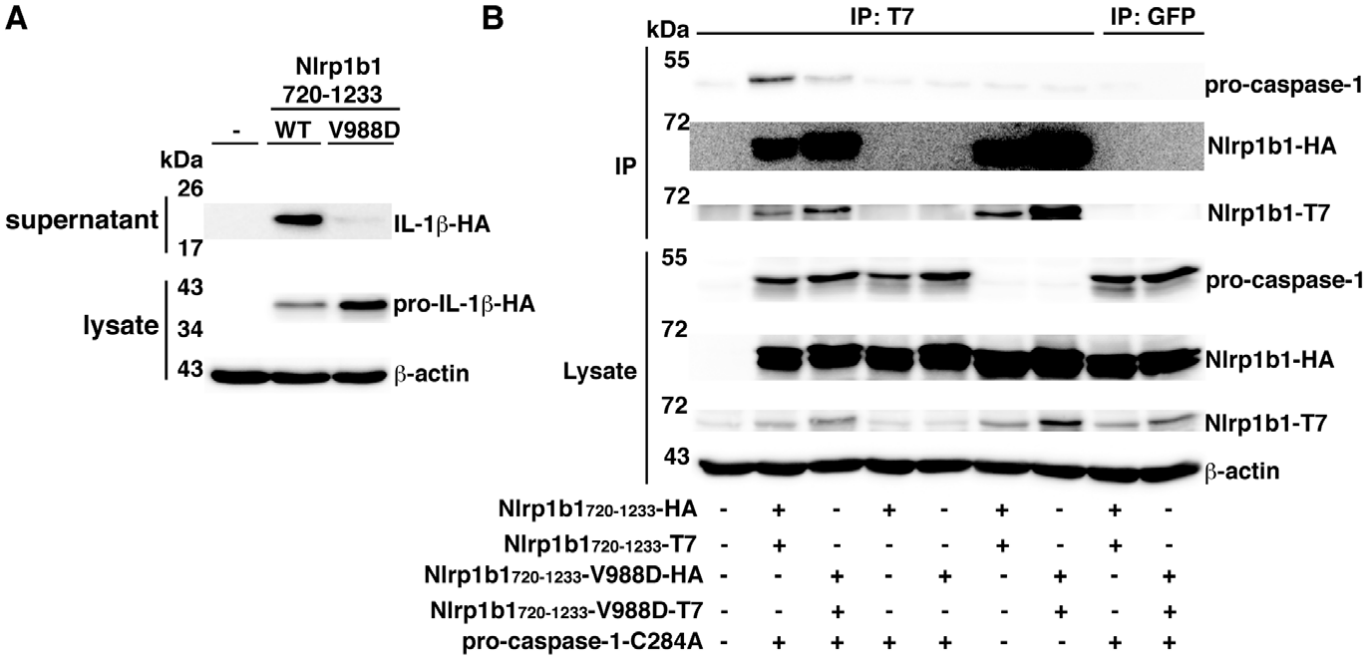
The V988D mutation in Nlrp1b1_720–1233_ reduces its ability to associate with pro-caspase-1. (A) HT1080 cells were transfected with pcDNA3-pro-caspase-1-T7 and pcDNA3-pro-IL-1β-HA and co-transfected as indicated with pNTAP-Nlrp1b1_720–1233_-HA, pNTAP-Nlrp1b1_720–1233_-T7, pNTAP-Nlrp1b1_720–1233_-V988D-HA, and pNTAP-Nlrp1b1_720–1233_-V988D. Approximately 24 h after transfection, cell lysates were collected and probed for HA-tagged pro-IL-1β and β-actin; cell supernatants were collected and immunoprecipitated with anti-HA antibodies and probed for HA-tagged IL-1β by immunoblotting. (B) Cells were transfected with indicated combinations of pNTAP-Nlrp1b1_720–1233_-HA and pNTAP-Nlrp1b1_720–1233_-T7 WT or V988D as well as with pcDNA3-pro-caspase-1-C284A-FLAG and pcDNA3-pro-IL-1β-HA. Approximately 24 h following transfection, cells were lysed and immunoprecipitated using an anti-T7 antibody. Proteins were immunoblotted with antibodies against HA, caspase-1, T7 and β-actin. Blots are representative of three independent experiments.

We next sought to determine how cleavage within the FIIND promotes inflammasome assembly. To ascertain whether processing within the FIIND facilitates the ability of the FIIND-CARD fragment to self-associate or to recruit pro-caspase-1, we used the constructs Nlrp1b1_720–1233_ and Nlrp1b1_720–1233_-V988D, which contain the entire FIIND-CARD region. Nlrp1b1_720–1233_ displayed constitutive activity as we have shown previously [11], but the V988D mutant had minimal activity (Figure 8A). An experiment was then performed in which T7-tagged Nlrp1b1 constructs were immunoprecipitated and the co-precipitation of HA-tagged Nlrp1b1 constructs and FLAG-tagged pro-caspase-1-C284A was tested. Nlrp1b1_720–1233_ self-associated in the presence or absence of pro-caspase-1-C284A (Figure 8B). Nlrp1b1_720–1233_ also complexed with pro-caspase-1-C284A. Nlrp1b1_720–1233_-V988D exhibited a slightly higher level of self-association compared to the wild-type construct, but bound less pro-caspase-1-C284A. These experiments suggest that cleavage within the FIIND facilitates the formation of an optimally assembled platform that can recruit pro-caspase-1.

## Discussion

NLRP1 and Nlrp1b appear to be unique among the NOD-like receptors in that they possess two regions that facilitate oligomerization: a NACHT domain and a FIIND-CARD region. The NACHT domain is a member of the well-characterized AAA+ ATPase family [27], while there is only limited information available on the FIIND. In this study, we discovered that cleavage of the Nlrp1b FIIND is functionally important for inflammasome activity and that this processing of the FIIND facilitates the recruitment of pro-caspase-1.

Previously, we noted that immunblotting for Nlrp1b1 using an antibody against the N-terminal TAP tag detected two bands [11]. We speculated that the upper band represented the full-length protein and that the lower band resulted from a proteolytic event. By generating a construct with tags at both termini of Nlrp1b1, we were able to detect the C-terminal fragment and determine that it remained associated with the N-terminal fragment (Figure 7). That the processing of Nlrp1b1 might be important for function was hinted at by the finding that the LeTx-non-responsive Nrlp1b3 was not cleaved. Furthermore, a substitution mutation that we designed to impair self-association of the FIIND unexpectedly prevented Nlrp1b1 cleavage. The functional importance of FIIND cleavage was established by an experiment in which the TEV protease site was introduced at a position within Nlrp1b1 that we estimated to be near the natural cleavage site – TEV protease cleaved this site and activated the inflammasome.

During the preparation of this manuscript, D'Osualdo and colleagues published that the FIINDs of human NLRP1 and CARD8 undergo autoproteolytic processing [23]. These researchers used computational approaches to detect similarity between the FIIND and the ZU5-UPA domains found in the PIDD autoprotease. PIDD forms a complex called the PIDDosome that either activates pro-casapase-2 or NF-κb in response to DNA damage. PIDD that is autoproteolytically cleaved at a single site initiates activation of NF-κb; an additional autoproteolytic event facilitates activation of pro-caspase-2 [24]. Autoproteolysis at each site occurs before a serine residue – the hydroxyl group of the serine is believed to attack a strained backbone to initiate the breakage of the peptide bond.

Cleavage of the CARD8 FIIND was demonstrated to occur after a phenylalanine residue in a conserved SFS motif [23], which corresponds to _982_SFS_984_ in Nlrp1b1. Notably, we found that the nearby V988D mutation in Nlrp1b1 abolished cleavage and the corresponding swap mutation in Nlrp1b3 caused this protein to be cleaved. Furthermore, the TEV site that allowed activation of Nlrp1b1-V988D-TEV by TEV protease was inserted between amino acids 981 and 982. Collectively, these findings suggest that the V988D mutation disrupts the protein conformation required for autoproteolysis between amino acids 983 and 984, but that activity can be rescued by cleavage at a nearby location. We believe that Nlrp1b1 undergoes autoproteolysis by the same mechanism as CARD8 because Nlrp1b1-S984A, in which the predicted catalytic serine is mutated, is not cleaved and is not functionally active (Figure S1). We further note that although lethal factor is itself a protease, the toxin does not affect Nlrp1b1 cleavage and, therefore, we do not believe that Nlrp1b1 is a direct target.

Only approximately half of overexpressed Nlrp1b1 and endogenous Nlrp1b1 is cleaved. We did not observe an increased amount of cleavage over time (data not shown), which suggests that only a fraction of Nlrp1b1 is capable of undergoing autoproteolysis. We speculate, therefore, that auto-inhibited Nlrp1b1 exists as a dimer in which one of the monomers is cleaved and the other is not cleaved. Dimerization might induce autoproteolysis in one of the monomers and not in the other, which could explain why mutation of amino acids 1100–1106 not only prevents self-association of Nlrp1b1_1100–1233_, but also prevents cleavage of Nlrp1b1.

To address the mechanistic consequence of FIIND processing, we compared Nlrp1b1_720–1233_ to Nlrp1b1_720–1233_-V988D, which contain the complete FIIND and CARD domains. Nlrp1b1_720–1233_ is cleaved and is constitutively active; the V988D mutation abolishes both cleavage and activity. We found that the V988D mutation does not impair self-association – and in fact seems to increase self-association to a small extent – but the mutation does impair the recruitment of pro-caspase-1. We interpret these data to mean that cleavage of the FIIND is required for the proper assembly of a platform that is capable of recruiting molecules of pro-caspase-1 in orientations that allow cross-proteolysis. Incorrect assembly of uncleaved FIIND-CARD may partially occlude the pro-caspase-1 binding site because self-association of FIIND-CARD does not appear to be required for binding pro-caspase-1, as demonstrated by binding of pro-caspase-1 to oligomerization-defective Nlrp1b1_1100–1233_-7A. Because Nlrp1b1_1100–1233_ is active even though it lacks the region that undergoes proteolysis, it is presumably the uncleaved region N-terminal to amino acids 1100–1233 that exerts a negative restraint that prevents correct assembly.

## Materials and Methods

### Cell lines and toxin

HT1080 cells and J774A.1 cells (ATCC) were cultured in Dulbecco's modified Eagle's medium supplemented with 10% fetal bovine serum and 1% penicillin-streptomycin. PA and LF were purified as described previously [28].

### Antibodies

Rabbit antibody was raised against an N-terminal epitope of Nlrp1b1, MEESPPKQKSNTKVAQHE. Membranes were probed with the following antibodies: anti-Nlrp1b polyclonal antibody (1∶5000), anti-caspase-1 p10 (M20) polyclonal antibody (1∶500, Santa Cruz Biotechnology sc-514), anti-HA polyclonal antibody (1∶1000, Santa Cruz Biotechnology sc-805), anti-T7-tag monoclonal antibody (1∶1000, Novagen 69522), anti-β-actin monoclonal antibody (1∶10000, Sigma Aldrich A-5441), and anti-calmodulin binding peptide (CBP) antibody (1∶1000, Upstate 07-482). For immunoprecipitations, anti-T7-tag monoclonal antibody (Novagen 69522), and anti-HA monoclonal antibody (Sigma H9658) were used. Anti-green fluorescent protein (GFP, Covance MMS-118R) was used as a control.

### Plasmid construction and site-directed mutagenesis

Construction of plasmids pcDNA3-T7, pcDNA3-His_6_-HA, pcDNA3-His_6_-T7, pN-TAPA Nlrp1b1, pcDNA3-pro-caspase-1-T7, pcDNA3-IL-1β-HA were described previously [11]. All substitution mutations were made using Quikchange mutagenesis (Stratagene).

To construct pcDNA3-pro-caspase-1-FLAG, pcDNA3-pro-caspase-1-T7 was cut with Apa1 and Nhe1 to remove the T7 tag. FLAG-tag oligonucleotide was constructed by annealing forward oligonucleotide 5′-CAT GGA CTA CAA GGA CGA CGA TGA CAA GG-3′ and reverse oligonucleotide 5′-CTA GCC TTG TCA TCG TCG TCC TTG TAG TCC ATG GGC C-3′. The resulting annealed oligonucleotide was ligated at Apa1 and NheI restriction sites of the restriction digested pcDNA3-pro-caspase-1-T7 vector.

pN-TAPB-T7 plasmid was constructed by annealing T7-tag forward oligonucleotide 5′- TCG AGA TGG CTA GCA TGA CTG GTG GAC AGC AAA TGG GTT AGG GGC C-3′ and reverse oligonucleotide 5′-CCT AAC CCA TTT GCT GTC CAC CAG TCA TGC TAG CCA TC-3′. The resulting annealed T7 oligonucleotide was ligated at Xho1 and Apa1 restriction sites of pN-TAPB.

Nlrp1b truncation plasmids were constructed by amplifying fragments from pN-TAPA -Nlrp1b1. The PCR products were digested with the restriction enzymes BamHI and XhoI, and the resulting products were ligated into vectors pN-TAPB-T7, pcDNA3-His_6_-HA and pcDNA3-His_6_-T7.

pC-TAPA-Nlrp1b1 was constructed by amplifying Nlrp1b1 with the forward primer 5′-GCG GGA TCC GCC GCC ACC ATG GAA GAA TCC CCA CCC AAG-3′ and the reverse primer 5′-GCG CTC GAG TGA TCC CAA AGA GAC CCC AC-3′. The PCR product was digested with BamHI and XhoI and ligated into pC-TAPA.

pN-TAPA-Nlrp1b1-TAP was constructed by amplifying the C-terminus of Nlrp1b1 and the TAP tag from the pC-TAPA-Nlrp1b1 plasmid using the forward primer 5′-CGC ACC CAA GCT TCT CCC CAA TGG-3′ and the reverse primer 5′-CGC CTC GAG CTA AAG TGC CCC GGA GGA TG-3′. The PCR product and pN-TAPA-Nlrp1b1 were digested with HindIII and XhoI. The vector backbone of pN-TAPA including the N-terminus of Nlrp1b1 were isolated by gel extraction (Qiagen) and ligated with the PCR product.

The NIa protease of the tobacco etch virus (TEV protease) was amplified using the forward primer 5′-CGC GGT ACC GCC GCC ACC ATG GGA TCC AGC TTG TTT AAG GGA C-3′ and the reverse primer 5′-CGC TCT AGA GTC ACG ATG AAT TCC GGG CGA G-3′. The PCR product was digested with KpnI and XbaI and ligated into pcDNA3-T7. In order to reduce the self-cleavage and increase the catalytic efficiency of TEV protease a S219V mutation [29] was introduced using the forward primer 5′-GGG GCC ATA AAG TTT TCA TGG TCA AAC CTG AAG AGC CTT TTC-3′ and the reverse primer 5′-GAA AAG GCT CTT CAG GTT TGA CCA TGA AAA CTT TAT GGC CCC-3′.

The pN-TAPA-Nlrp1b1-V988D-TEV construct was cloned in two steps. The N-terminus of Nlrp1b1 was amplified with the forward primer 5′-CGC GGA TCC TAT GGA AGA ATC CCC ACC CAA G-3′ and the reverse primer (which includes coding for the TEV-site) 5′-CGC ATC GTC GAC TGG AAG TAG AGA TTC TCT GGG TTT TTC AGT ACT GTG TAT CC-3′. The C-terminus of Nlrp1b1 was amplified from pNTAP-Nlrp1b1-V988D with the forward primer 5′-CGC ATC GTC GAC GAG CTT CTC CCC AAT GGG AGA TG-3′ and the reverse primer 5′-CGC CTC GAG TCA TGA TCC CAA AGA GAC CCC ACC TG-3′. The PCR fragments were digested with SalI and ligated. After gel extraction, the ligated PCR fragments were digested with BamHI and XhoI and ligated into pN-TAPA.

To construct pcDNA3-His_6_-Nlrp1b1-HA, Nlrp1b1 was amplified using the forward primer 5′-CGC GGA TCC ATG GAA GAA TCC CCA CCC AAG-3′ and the reverse primer 5′- CGC CTC GAG TGA TCC CAA AGA GAC CCC AC-3′. The PCR product was digested using BamHI and XhoI followed by ligation into pcDNA3-His_6_-HA.

The pN-TAPA-Nlrp1b1-HA construct was created by amplifying Nlrp1b1 including the C-terminal HA tag from pcDNA3- His_6_-Nlrp1b-HA. The forward primer used was 5′-CGC GGA TCC TAT GGA AGA ATC CCC ACC CAA G-3′ and the reverse primer was 5′-CGC ATC GTG GTC GAC TCA CAA GCT AGC GTA ATC TGG-3′. The PCR product was digested with BamHI and SalI and ligated into pN-TAPA.

pN-TAPB-Nlrp1b1_720–1233_-T7 was constructed by amplifying Nlrp1b1 using the forward primer 5′-GCG GGA TCC GAC CTG TCC TCT CTC AGT G-3′ and the reverse primer 5′-CGC CTC GAG TGA TCC CAA AGA GAC CCC AC-3′. The PCR product was digested with BamHI and XhoI and ligated into pN-TAPB-T7.

The pN-TAPB-Nlrp1b1_720–1233_-HA construct was created by amplifying the FIIND and CARD domains of Nlrp1b1 including the C-terminal HA tag from pcDNA3- His_6_-Nlrp1b-HA. The forward primer used was 5′-GCG GGA TCC GAC CTG TCC TCT CTC AGT GCC-3′ and the reverse primer was 5′-CGC ATC GTG GTC GAC TCA CAA GCT AGC GTA ATC TGG-3′. The PCR product was digested with BamHI and SalI and ligated into pN-TAPB.

### Nlrp1b1 knockdown with shRNA

Design and infection of shRNA were performed according to pLKO.1 protocol from Addgene. Nlrp1b1 shRNA was targeted against the 3′ UTR of Nlrp1b1. The Nlrp1b1 shRNA forward sequence was 5′-CCG GGG TTG TCT TTG TCT CTG TTG ACT CGA GTC AAC AGA GAC AAA GAC AAC C-3′ and the reverse sequence was 5′-AAT TCA AAA AGG TTG TCT TTG TCT CTG TTG ACT CGA GTC AAC AGA GAC AAA GAC AAC C-3′. The scrambled shRNA sequences were generated from the Nlrp1b1 shRNA sequence and the forward sequence was 5′-CCG GGG TCT TGT ATT CGG TTT CTG TCT CGA GAC AGA AAC CGA ATA CAA GAC CTT TTT G-3′ while the reverse sequence was 5′-AAT TCA AAA AGG TCT TGT ATT CGG TTT CTG TCT CGA GAC AGA AAC CGA ATA CAA GAC C-3′. Forward and reverse oligonucleotides were annealed and ligated into the pLKO.1 vector at the EcoRI and AgeI sites. pLKO.1 vector containing the shRNA was transfected with polyethylenimine, pH 7.2 into HEK-293T cells (ATCC). Approximately 48 h following transfection, cell medium was collected and filtered with a 0.45 µm filter and 2 mL of the lentiviral particle solution was added to a 10 cm dish containing J774A.1 cells. Approximately 24 h later, media was removed and media containing 5 µg/ml puromycin (Sigma) was added to J774A.1 cells to select cells that had stably integrated the shRNA expressing plasmid.

### Lactate dehydrogenase release assay

J774A.1 cells were seeded onto a 24-well plate at 1.4×10^5^ cells per well. Approximately 24 h later cells were treated with LeTx (10^−8^ M PA and 10^−9^ M LF) for 4 h. Release of cytoplasmic LDH into the cell medium was measured using the CytoTox 96 nonradioactive cytotoxicity assay (Promega G-1780) in accordance with the manufacturer's instructions. The percent of LDH release was calculated as 100×(Experimental LDH−Spontaneous LDH)/(Maximum LDH−Spontaneous LDH). J774A.1 WT −LeTx and +LeTx values were set to 0% and 100% respectively, and scrambled and Nlrp1b shRNA J774A.1 results were normalized to those values. Results from three independent experiments were averaged.

### IL-1β release assay

Approximately one million HT1080 cells were seeded on a 10-cm dish the day before transfection. On the day of transfection, 1 µg each of pN-TAP-Nlrp1b1, pcDNA3-pro-caspase-1-FLAG (or T7), and pcDNA3-pro-IL-1β-HA was transfected using 9 µl of 1 mg/ml polyethylenimine, pH 7.2. Note that for experiments involving TEV protease an additional 1 µg of either pcDNA3-TEV-protease-T7 or pcDNA3 empty vector was transfected as indicated. Approximately 24 h after transfection, cells were treated with LF (10^−8^ M) and PA (10^−8^ M) for 3 h. The cell supernatant was mixed with 1 µl of anti- HA antibody (Sigma-Aldrich H9658) overnight, followed by the addition of 100 µl of BSA-blocked protein A Sepharose beads (GE Healthcare) and a 2 h incubation. Proteins were eluted from the protein A Sepharose beads with sodium dodecyl sulfate (SDS) loading dye and subjected to immunoblotting using a polyclonal HA antibody (Santa Cruz sc805). Cells from each 10-cm plate were scraped into 300 µl of EBC lysis buffer (50 mM Tris, pH 8, 150 mM NaCl, 0.5% [vol/vol] NP-40, 1 mM phenylmethylsulfonyl fluoride). Each sample was lysed by rotation at 4°C for 1 h or by sonication 3 times, each for 10 s followed by a 10 s incubation on ice. Lysates were clarified by centrifugation, and protein concentrations were determined using the Bradford assay. Equivalent amounts of cell lysate protein (∼40 µg) were subjected to SDS-polyacrylamide gel electrophoresis and immunoblotted with anti-HA, anti-T7, and anti-β-actin antibodies.

### Detection of TAP-tagged proteins

One to five 10-cm dishes of HT1080 cells were transfected with 1–4 µg of the indicated constructs. Approximately 24 h after transfection, cells were harvested and cell pellets from each plate were lysed with approximately 300 µl EBC buffer by sonication. Cell lysates were clarified by centrifugation. Equivalent amount of cell lysate protein was incubated with 25 or 50 µL streptavidin agarose resin (Thermo Scientific 20349) for ∼2 h or overnight. Beads were washed three times with 1 mL EBC buffer. Proteins were eluted with SDS and analyzed by immunoblotting with anti-Nlrp1b or anti-CBP antibody. CBP is an epitope found within the TAP tag.

### Co-immunoprecipitation assays

To test self-association of Nlrp1b1 truncation mutants, two plates of HT1080 cells were transfected with pcDNA3-His_6_-Nlrp1b1-HA and pcDNA3-His_6_-Nlrp1b1-T7 vectors containing trunction mutants. Approximately 24 h following transfection, cells were lysed in 300 µL EBC buffer by sonication, and lysates were clarified by centrifugation. Equal amount of lysate protein was incubated overnight with 1 µL of anti-HA antibody (Sigma-Aldrich H9658), followed by the addition of 50 µL of BSA-blocked protein A Sepharose beads (GE Healthcare) and a 2 h incubation at 4°C. Complexes were resolved by SDS-polyacrylamide gel electrophoresis and immunoblotted using an anti-T7 antibody.

A similar protocol as above was followed to test for Nlrp1b1_720–1233_ self-association and pro-caspase-1 binding, with the exception that the vectors indicated in Figure 8 were transfected to a total of 4 µg. Lysates were incubated with 1 µl of anti-T7 antibody or 1 µL of control anti-GFP antibody overnight. Membranes were immunoblotted with anti-caspase-1 p10, anti-HA, anti-T7, and anti-β-actin antibodies.

A similar protocol as above was followed to test for the association of the Nlrp1b1 cleaved products with the exception that the vectors indicated in Figure 7 were transfected to a total of 4 µg. Lysates were incubated overnight with 50 µL streptavidin agarose resin slurry, followed by washing three times with 1 mL EBC buffer. Proteins were eluted from the beads with SDS and analyzed by immunoblotting with anti-HA and anti-β-actin antibodies.

For the experiment shown in Figure 3C, two 10-cm dishes of HT1080 cells were transfected with pcDNA3-pro-caspase-1-T7-C284A and either pcDNA3-His_6_-Nlrp1b1_1100–1233_-HA, pcDNA3-His_6_-Nlrp1b1_1100–1233_-7A-HA or pcDNA3-His_6_-Nlrp1b1_1100–1233_-HA. Cells were lysed in 600 µL EBC buffer by sonication, and the lysates were clarified by centrifugation. Lysates were incubated with 1 µL of anti-T7 antibody or 1 µL of control anti-GFP antibody overnight, followed by the addition of 50 µL of protein A Sepharose for 2 h. Complexes were resolved by SDS-polyacrylamide gel electrophoresis and immunoblotted using an anti-HA antibody and anti-caspase-1 p10 antibody.

## Supporting Information

Figure S1 **The S984A mutation in Nlrp1b1 eliminates both cleavage and inflammasome activity.** (A) HT1080 cells were transfected with pNTAP plasmids encoding the indicated Nlrp1b proteins. Approximately 24 h following transfection, cells were lysed and TAP-tagged proteins were precipitated with streptavidin resin and immunoblotted using an Nlrp1b antibody. (B) Cells were transfected with pNTAP plasmids encoding the indicated Nlrp1b constructs, as well as with pcDNA3-pro-caspase-1-T7 and pcDNA3-pro-IL-1β-HA. Approximately 24 h after transfection, cells were treated with LeTx (10^−8^ M LF and 10^−8^ M PA) for 3 h. Cell lysates were collected and probed for HA-tagged pro-IL-1β and β-actin; cell supernatants were collected and immunoprecipitated with anti-HA antibodies and probed for HA-tagged IL-1β by immunoblotting. Blots are representative of three independent experiments.
(TIF)
