# Supplementary figures and images for: Proteolytic Processing of Nlrp1b Is Required for Inflammasome Activity
Source: PLoS Pathog. 2012 Apr 19;8(4):e1002659. doi: 10.1371/journal.ppat.1002659 (PMC3334886; doi:10.1371/journal.ppat.1002659)

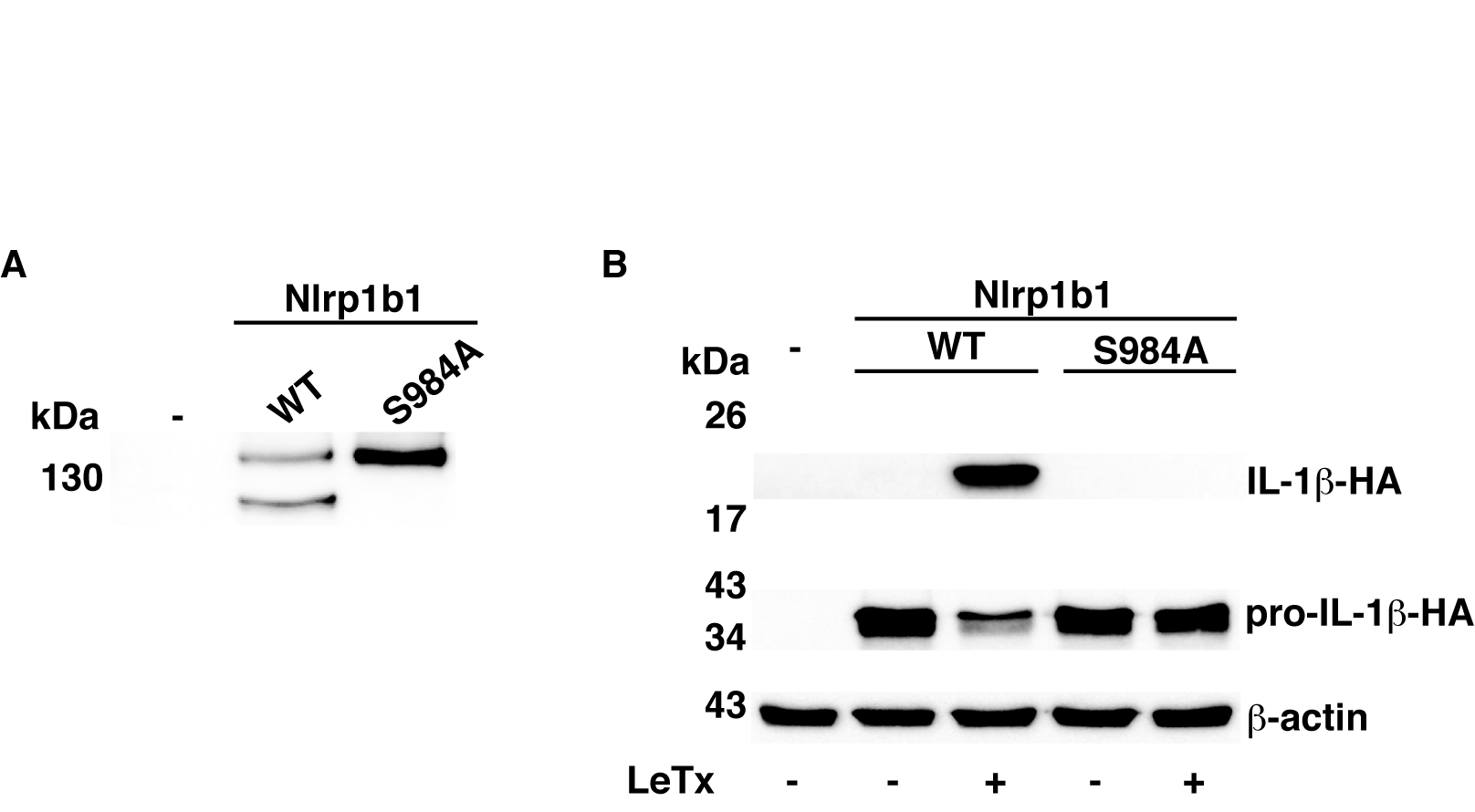

Supplement: Figure S1 — The S984A mutation in Nlrp1b1 eliminates both cleavage and inflammasome activity. (A) HT1080 cells were transfected with pNTAP plasmids encoding the indicated Nlrp1b proteins. Approximately 24 h following transfection, cells were lysed and TAP-tagged proteins were precipitated with streptavidin resin and immunoblotted using an Nlrp1b antibody. (B) Cells were transfected with pNTAP plasmids encoding the indicated Nlrp1b constructs, as well as with pcDNA3-pro-caspase-1-T7 and pcDNA3-pro-IL-1β-HA. Approximately 24 h after transfection, cells were treated with LeTx (10−8 M LF and 10−8 M PA) for 3 h. Cell lysates were collected and probed for HA-tagged pro-IL-1β and β-actin; cell supernatants were collected and immunoprecipitated with anti-HA antibodies and probed for HA-tagged IL-1β by immunoblotting. Blots are representative of three independent experiments. (TIF) [file ppat.1002659.s001.tif]
